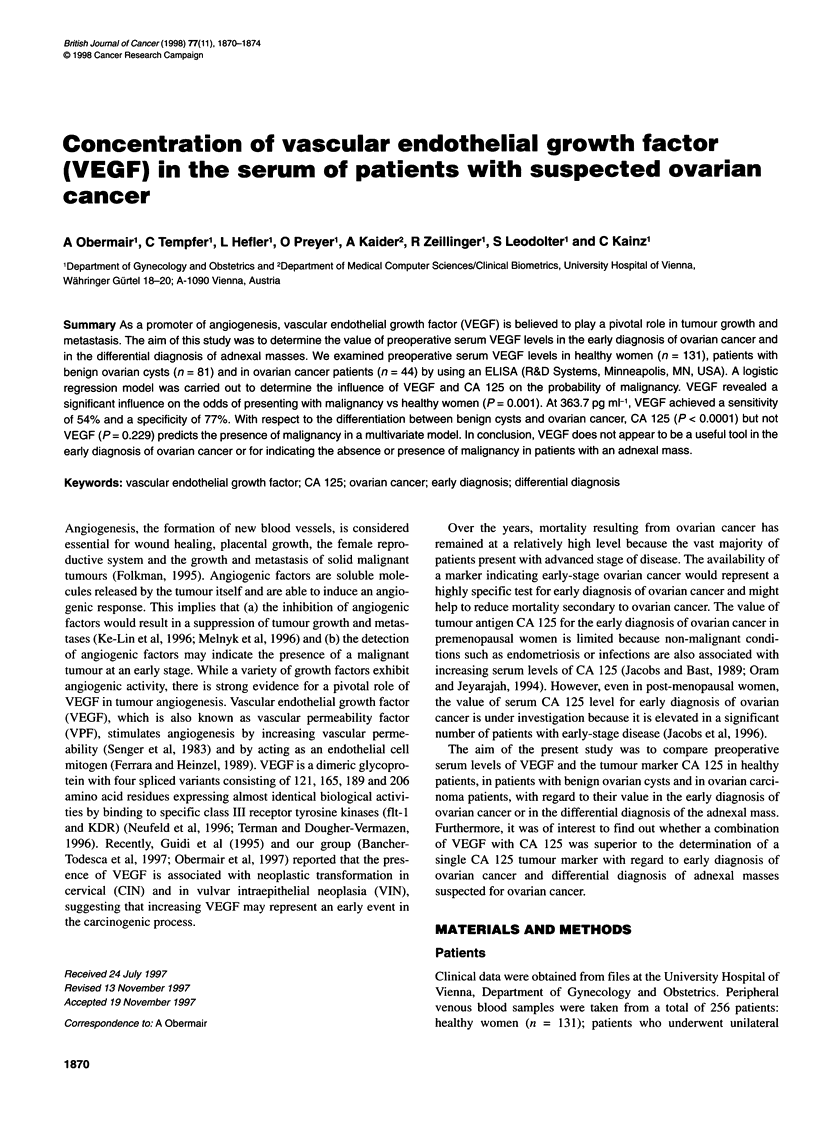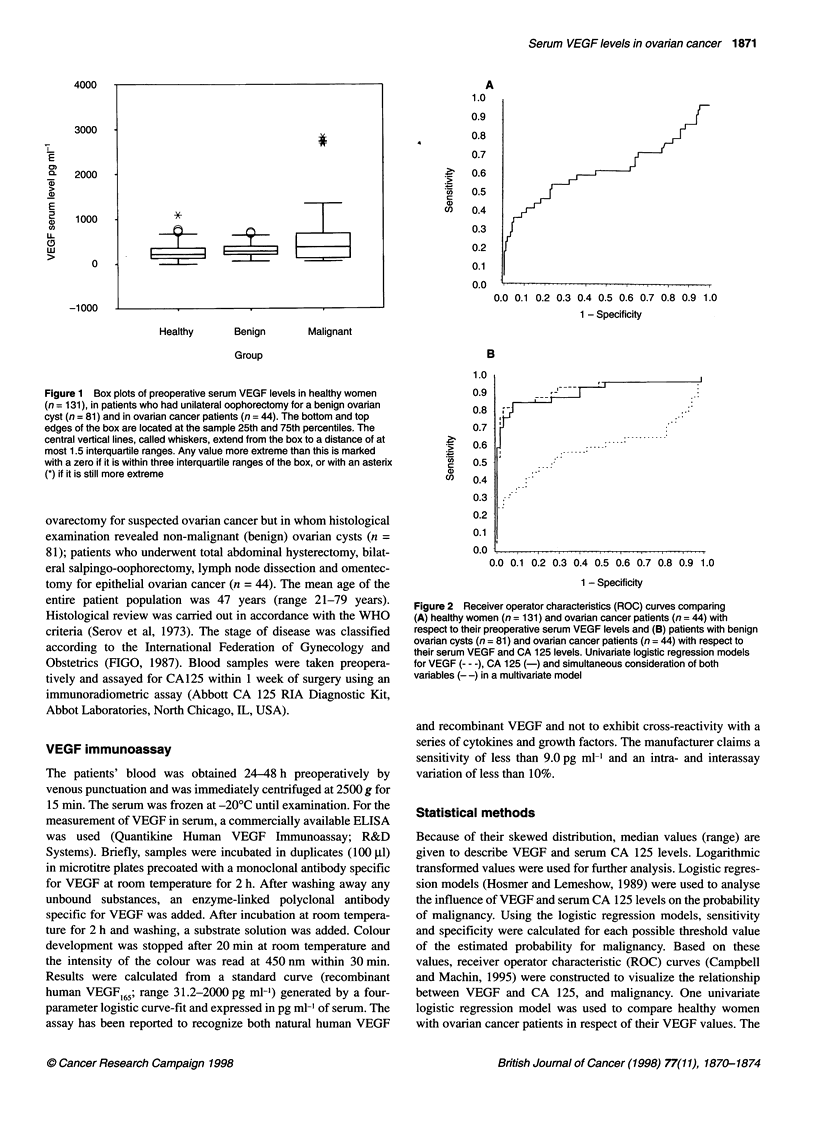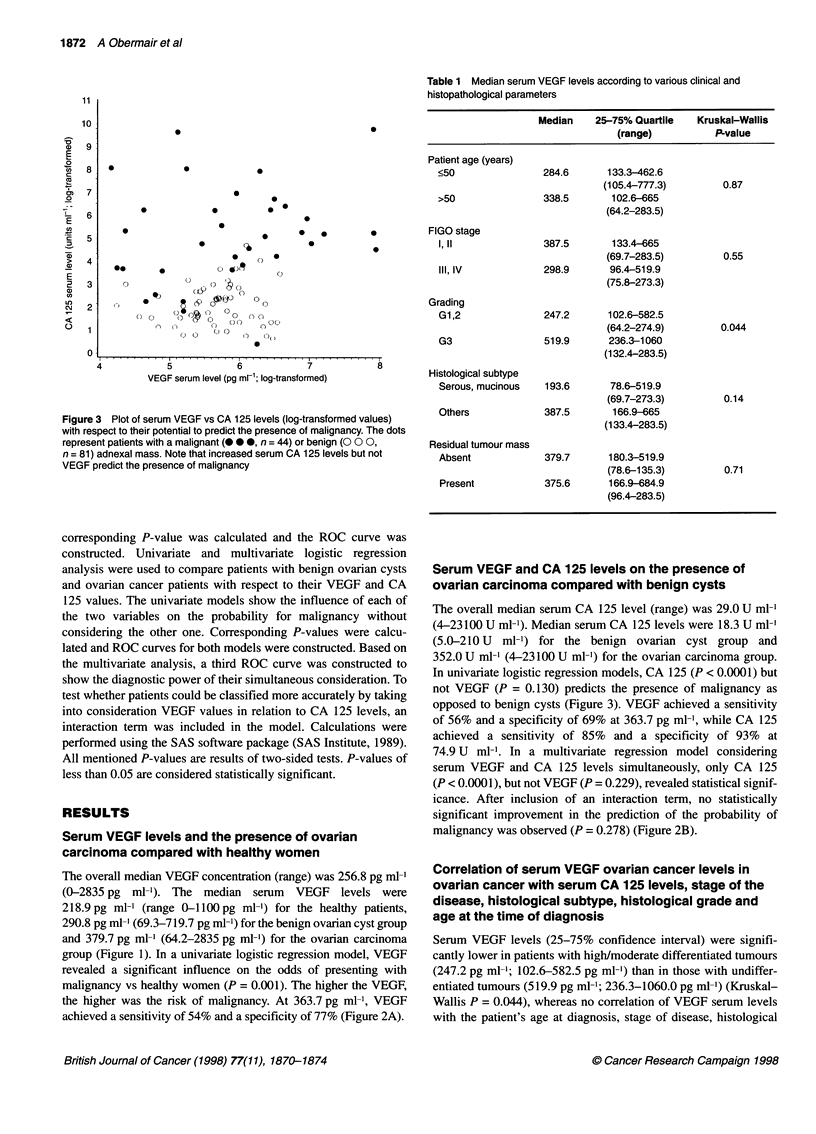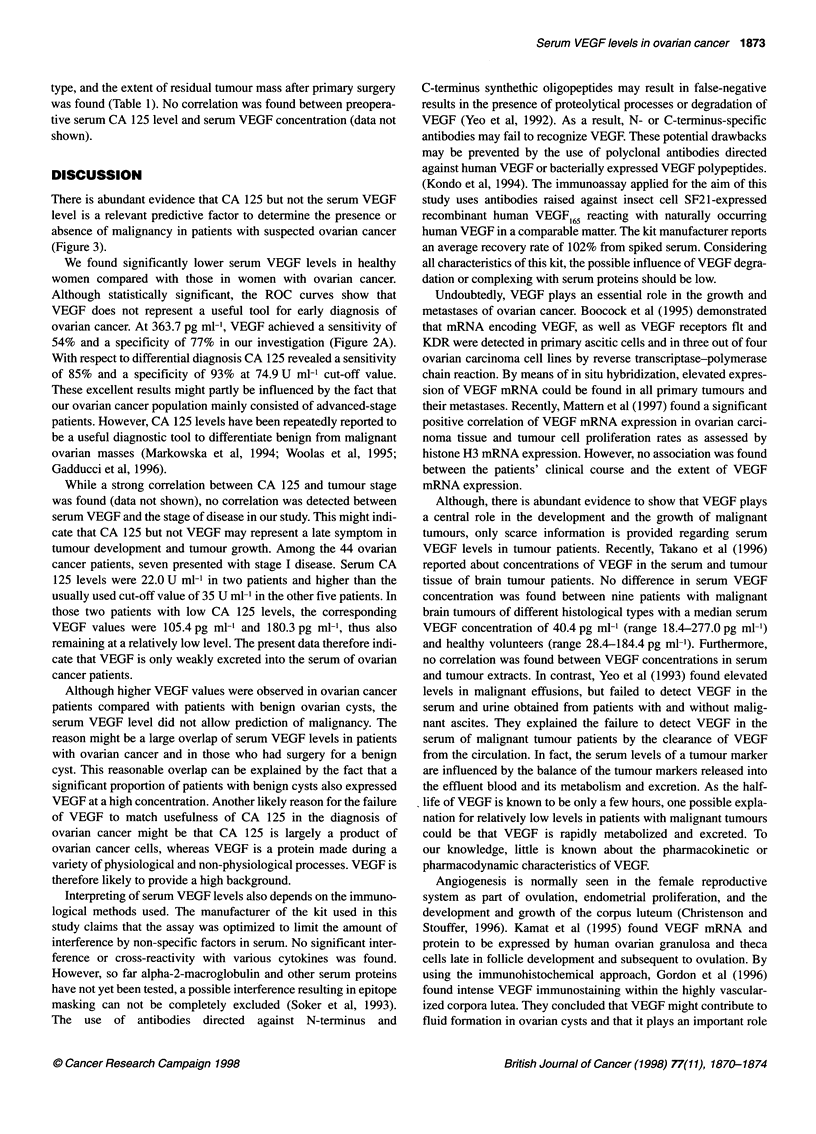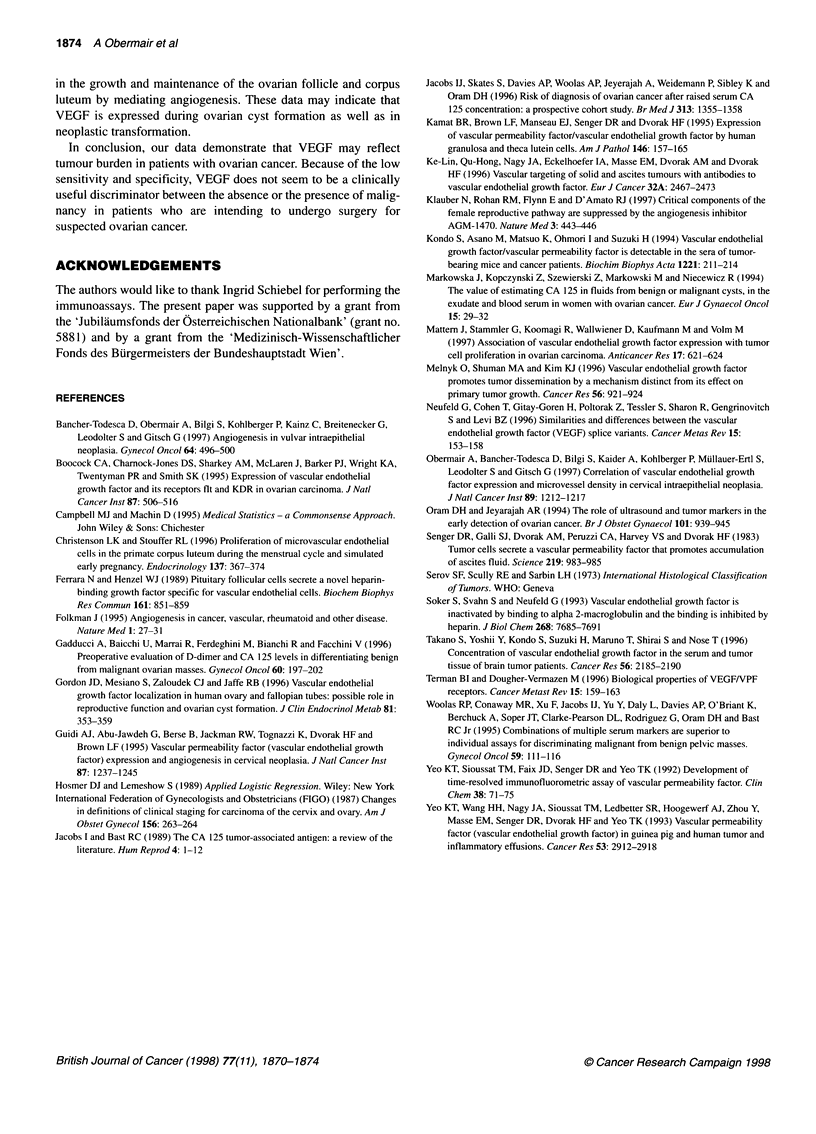# Concentration of vascular endothelial growth factor (VEGF) in the serum of patients with suspected ovarian cancer.

**DOI:** 10.1038/bjc.1998.311

**Published:** 1998-06

**Authors:** A. Obermair, C. Tempfer, L. Hefler, O. Preyer, A. Kaider, R. Zeillinger, S. Leodolter, C. Kainz

**Affiliations:** Department of Gynecology and Obstetrics, University Hospital of Vienna, Austria.

## Abstract

As a promoter of angiogenesis, vascular endothelial growth factor (VEGF) is believed to play a pivotal role in tumour growth and metastasis. The aim of this study was to determine the value of preoperative serum VEGF levels in the early diagnosis of ovarian cancer and in the differential diagnosis of adnexal masses. We examined preoperative serum VEGF levels in healthy women (n = 131), patients with benign ovarian cysts (n = 81) and in ovarian cancer patients (n = 44) by using an ELISA (R&D Systems, Minneapolis, MN, USA). A logistic regression model was carried out to determine the influence of VEGF and CA 125 on the probability of malignancy. VEGF revealed a significant influence on the odds of presenting with malignancy vs healthy women (P = 0.001). At 363.7 pg ml(-1), VEGF achieved a sensitivity of 54% and a specificity of 77%. With respect to the differentiation between benign cysts and ovarian cancer, CA 125 (P < 0.0001) but not VEGF (P = 0.229) predicts the presence of malignancy in a multivariate model. In conclusion, VEGF does not appear to be a useful tool in the early diagnosis of ovarian cancer or for indicating the absence or presence of malignancy in patients with an adnexal mass.


British Journal of Cancer (1998) 77(11), 1870–1874
© 1998 Cancer Research Campaign

### Concentration of vascular endothelial growth factor (VEGF) in the serum of patients with suspected ovarian cancer

**A Obermair[1], C Tempfer[1], L Hefler[1], O Preyer[1], A Kaider[2], R Zeillinger[1], S Leodolter[1] and C Kainz[1]**

[1]Department of Gynecology and Obstetrics and [2]Department of Medical Computer Sciences/Clinical Biometrics, University Hospital of Vienna, Währinger Gürtel 18–20; A-1090 Vienna, Austria

**Summary** As a promoter of angiogenesis, vascular endothelial growth factor (VEGF) is believed to play a pivotal role in tumour growth and metastasis. The aim of this study was to determine the value of preoperative serum VEGF levels in the early diagnosis of ovarian cancer and in the differential diagnosis of adnexal masses. We examined preoperative serum VEGF levels in healthy women ($n$ = 131), patients with benign ovarian cysts ($n$ = 81) and in ovarian cancer patients ($n$ = 44) by using an ELISA (R&D Systems, Minneapolis, MN, USA). A logistic regression model was carried out to determine the influence of VEGF and CA 125 on the probability of malignancy. VEGF revealed a significant influence on the odds of presenting with malignancy vs healthy women ($P = 0.001$). At 363.7 pg ml$^{-1}$, VEGF achieved a sensitivity of 54% and a specificity of 77%. With respect to the differentiation between benign cysts and ovarian cancer, CA 125 ($P < 0.0001$) but not VEGF ($P = 0.229$) predicts the presence of malignancy in a multivariate model. In conclusion, VEGF does not appear to be a useful tool in the early diagnosis of ovarian cancer or for indicating the absence or presence of malignancy in patients with an adnexal mass.

**Keywords:** vascular endothelial growth factor; CA 125; ovarian cancer; early diagnosis; differential diagnosis

Angiogenesis, the formation of new blood vessels, is considered essential for wound healing, placental growth, the female reproductive system and the growth and metastasis of solid malignant tumours (Folkman, 1995). Angiogenic factors are soluble molecules released by the tumour itself and are able to induce an angiogenic response. This implies that (a) the inhibition of angiogenic factors would result in a suppression of tumour growth and metastases (Ke-Lin et al, 1996; Melnyk et al, 1996) and (b) the detection of angiogenic factors may indicate the presence of a malignant tumour at an early stage. While a variety of growth factors exhibit angiogenic activity, there is strong evidence for a pivotal role of VEGF in tumour angiogenesis. Vascular endothelial growth factor (VEGF), which is also known as vascular permeability factor (VPF), stimulates angiogenesis by increasing vascular permeability (Senger et al, 1983) and by acting as an endothelial cell mitogen (Ferrara and Heinzel, 1989). VEGF is a dimeric glycoprotein with four spliced variants consisting of 121, 165, 189 and 206 amino acid residues expressing almost identical biological activities by binding to specific class III receptor tyrosine kinases (flt-1 and KDR) (Neufeld et al, 1996; Terman and Dougher-Vermazen, 1996). Recently, Guidi et al (1995) and our group (Bancher-Todesca et al, 1997; Obermair et al, 1997) reported that the presence of VEGF is associated with neoplastic transformation in cervical (CIN) and in vulvar intraepithelial neoplasia (VIN), suggesting that increasing VEGF may represent an early event in the carcinogenic process.

*Received 24 July 1997*
*Revised 13 November 1997*
*Accepted 19 November 1997*

*Correspondence to:* A Obermair

Over the years, mortality resulting from ovarian cancer has remained at a relatively high level because the vast majority of patients present with advanced stage of disease. The availability of a marker indicating early-stage ovarian cancer would represent a highly specific test for early diagnosis of ovarian cancer and might help to reduce mortality secondary to ovarian cancer. The value of tumour antigen CA 125 for the early diagnosis of ovarian cancer in premenopausal women is limited because non-malignant conditions such as endometriosis or infections are also associated with increasing serum levels of CA 125 (Jacobs and Bast, 1989; Oram and Jeyarajah, 1994). However, even in post-menopausal women, the value of serum CA 125 level for early diagnosis of ovarian cancer is under investigation because it is elevated in a significant number of patients with early-stage disease (Jacobs et al, 1996).

The aim of the present study was to compare preoperative serum levels of VEGF and the tumour marker CA 125 in healthy patients, in patients with benign ovarian cysts and in ovarian carcinoma patients, with regard to their value in the early diagnosis of ovarian cancer or in the differential diagnosis of the adnexal mass. Furthermore, it was of interest to find out whether a combination of VEGF with CA 125 was superior to the determination of a single CA 125 tumour marker with regard to early diagnosis of ovarian cancer and differential diagnosis of adnexal masses suspected for ovarian cancer.

### MATERIALS AND METHODS

### Patients

Clinical data were obtained from files at the University Hospital of Vienna, Department of Gynecology and Obstetrics. Peripheral venous blood samples were taken from a total of 256 patients: healthy women ($n$ = 131); patients who underwent unilateral

**Figure 1** Box plots of preoperative serum VEGF levels in healthy women ($n$ = 131), in patients who had unilateral oophorectomy for a benign ovarian cyst ($n$ = 81) and in ovarian cancer patients ($n$ = 44). The bottom and top edges of the box are located at the sample 25th and 75th percentiles. The central vertical lines, called whiskers, extend from the box to a distance of at most 1.5 interquartile ranges. Any value more extreme than this is marked with a zero if it is within three interquartile ranges of the box, or with an asterix (*) if it is still more extreme

**Figure 2** Receiver operator characteristics (ROC) curves comparing (**A**) healthy women ($n$ = 131) and ovarian cancer patients ($n$ = 44) with respect to their preoperative serum VEGF levels and (**B**) patients with benign ovarian cysts ($n$ = 81) and ovarian cancer patients ($n$ = 44) with respect to their serum VEGF and CA 125 levels. Univariate logistic regression models for VEGF (- - -), CA 125 (—) and simultaneous consideration of both variables (– –) in a multivariate model ovarectomy for suspected ovarian cancer but in whom histological examination revealed non-malignant (benign) ovarian cysts ($n$ = 81); patients who underwent total abdominal hysterectomy, bilateral salpingo-oophorectomy, lymph node dissection and omentectomy for epithelial ovarian cancer ($n$ = 44). The mean age of the entire patient population was 47 years (range 21–79 years). Histological review was carried out in accordance with the WHO criteria (Serov et al, 1973). The stage of disease was classified according to the International Federation of Gynecology and Obstetrics (FIGO, 1987). Blood samples were taken preoperatively and assayed for CA125 within 1 week of surgery using an immunoradiometric assay (Abbott CA 125 RIA Diagnostic Kit, Abbot Laboratories, North Chicago, IL, USA).

### VEGF immunoassay

The patients' blood was obtained 24–48 h preoperatively by venous punctuation and was immediately centrifuged at 2500 ***g*** for 15 min. The serum was frozen at −20°C until examination. For the measurement of VEGF in serum, a commercially available ELISA was used (Quantikine Human VEGF Immunoassay; R&D Systems). Briefly, samples were incubated in duplicates (100 μl) in microtitre plates precoated with a monoclonal antibody specific for VEGF at room temperature for 2 h. After washing away any unbound substances, an enzyme-linked polyclonal antibody specific for VEGF was added. After incubation at room temperature for 2 h and washing, a substrate solution was added. Colour development was stopped after 20 min at room temperature and the intensity of the colour was read at 450 nm within 30 min. Results were calculated from a standard curve (recombinant human $VEGF_{165}$; range 31.2–2000 pg $ml^{-1}$) generated by a four-parameter logistic curve-fit and expressed in pg $ml^{-1}$ of serum. The assay has been reported to recognize both natural human VEGF and recombinant VEGF and not to exhibit cross-reactivity with a series of cytokines and growth factors. The manufacturer claims a sensitivity of less than 9.0 pg $ml^{-1}$ and an intra- and interassay variation of less than 10%.

### Statistical methods

Because of their skewed distribution, median values (range) are given to describe VEGF and serum CA 125 levels. Logarithmic transformed values were used for further analysis. Logistic regression models (Hosmer and Lemeshow, 1989) were used to analyse the influence of VEGF and serum CA 125 levels on the probability of malignancy. Using the logistic regression models, sensitivity and specificity were calculated for each possible threshold value of the estimated probability for malignancy. Based on these values, receiver operator characteristic (ROC) curves (Campbell and Machin, 1995) were constructed to visualize the relationship between VEGF and CA 125, and malignancy. One univariate logistic regression model was used to compare healthy women with ovarian cancer patients in respect of their VEGF values. The

© Cancer Research Campaign 1998

Figure 3 Plot of serum VEGF vs CA 125 levels (log-transformed values) with respect to their potential to predict the presence of malignancy. The dots represent patients with a malignant (● ● ●, $n = 44$) or benign (O O O, $n = 81$) adnexal mass. Note that increased serum CA 125 levels but not VEGF predict the presence of malignancy

Table 1 Median serum VEGF levels according to various clinical and histopathological parameters

| | Median | 25–75% Quartile (range) | Kruskal-Wallis *P*-value |
|---|---|---|---|
| Patient age (years) | | | |
| ≤50 | 284.6 | 133.3–462.6 (105.4–777.3) | 0.87 |
| >50 | 338.5 | 102.6–665 (64.2–283.5) | |
| FIGO stage | | | |
| I, II | 387.5 | 133.4–665 (69.7–283.5) | 0.55 |
| III, IV | 298.9 | 96.4–519.9 (75.8–273.3) | |
| Grading | | | |
| G1,2 | 247.2 | 102.6–582.5 (64.2–274.9) | 0.044 |
| G3 | 519.9 | 236.3–1060 (132.4–283.5) | |
| Histological subtype | | | |
| Serous, mucinous | 193.6 | 78.6–519.9 (69.7–273.3) | 0.14 |
| Others | 387.5 | 166.9–665 (133.4–283.5) | |
| Residual tumour mass | | | |
| Absent | 379.7 | 180.3–519.9 (78.6–135.3) | 0.71 |
| Present | 375.6 | 166.9–684.9 (96.4–283.5) | |

corresponding *P*-value was calculated and the ROC curve was constructed. Univariate and multivariate logistic regression analysis were used to compare patients with benign ovarian cysts and ovarian cancer patients with respect to their VEGF and CA 125 values. The univariate models show the influence of each of the two variables on the probability for malignancy without considering the other one. Corresponding *P*-values were calculated and ROC curves for both models were constructed. Based on the multivariate analysis, a third ROC curve was constructed to show the diagnostic power of their simultaneous consideration. To test whether patients could be classified more accurately by taking into consideration VEGF values in relation to CA 125 levels, an interaction term was included in the model. Calculations were performed using the SAS software package (SAS Institute, 1989). All mentioned *P*-values are results of two-sided tests. *P*-values of less than 0.05 are considered statistically significant.

### RESULTS

### Serum VEGF levels and the presence of ovarian carcinoma compared with healthy women

The overall median VEGF concentration (range) was 256.8 pg $ml^{-1}$ (0–2835 pg $ml^{-1}$). The median serum VEGF levels were 218.9 pg $ml^{-1}$ (range 0–1100 pg $ml^{-1}$) for the healthy patients, 290.8 pg $ml^{-1}$ (69.3–719.7 pg $ml^{-1}$) for the benign ovarian cyst group and 379.7 pg $ml^{-1}$ (64.2–2835 pg $ml^{-1}$) for the ovarian carcinoma group (Figure 1). In a univariate logistic regression model, VEGF revealed a significant influence on the odds of presenting with malignancy vs healthy women ($P = 0.001$). The higher the VEGF, the higher was the risk of malignancy. At 363.7 pg $ml^{-1}$, VEGF achieved a sensitivity of 54% and a specificity of 77% (Figure 2A).

### Serum VEGF and CA 125 levels on the presence of ovarian carcinoma compared with benign cysts

The overall median serum CA 125 level (range) was 29.0 U $ml^{-1}$ (4–23100 U $ml^{-1}$). Median serum CA 125 levels were 18.3 U $ml^{-1}$ (5.0–210 U $ml^{-1}$) for the benign ovarian cyst group and 352.0 U $ml^{-1}$ (4–23100 U $ml^{-1}$) for the ovarian carcinoma group. In univariate logistic regression models, CA 125 ($P < 0.0001$) but not VEGF ($P = 0.130$) predicts the presence of malignancy as opposed to benign cysts (Figure 3). VEGF achieved a sensitivity of 56% and a specificity of 69% at 363.7 pg $ml^{-1}$, while CA 125 achieved a sensitivity of 85% and a specificity of 93% at 74.9 U $ml^{-1}$. In a multivariate regression model considering serum VEGF and CA 125 levels simultaneously, only CA 125 ($P < 0.0001$), but not VEGF ($P = 0.229$), revealed statistical significance. After inclusion of an interaction term, no statistically significant improvement in the prediction of the probability of malignancy was observed ($P = 0.278$) (Figure 2B).

### Correlation of serum VEGF ovarian cancer levels in ovarian cancer with serum CA 125 levels, stage of the disease, histological subtype, histological grade and age at the time of diagnosis

Serum VEGF levels (25–75% confidence interval) were significantly lower in patients with high/moderate differentiated tumours (247.2 pg $ml^{-1}$; 102.6–582.5 pg $ml^{-1}$) than in those with undifferentiated tumours (519.9 pg $ml^{-1}$; 236.3–1060.0 pg $ml^{-1}$) (Kruskal–Wallis $P = 0.044$), whereas no correlation of VEGF serum levels with the patient's age at diagnosis, stage of disease, histological

© Cancer Research Campaign 1998

type, and the extent of residual tumour mass after primary surgery was found (Table 1). No correlation was found between preoperative serum CA 125 level and serum VEGF concentration (data not shown).

### DISCUSSION

There is abundant evidence that CA 125 but not the serum VEGF level is a relevant predictive factor to determine the presence or absence of malignancy in patients with suspected ovarian cancer (Figure 3).

We found significantly lower serum VEGF levels in healthy women compared with those in women with ovarian cancer. Although statistically significant, the ROC curves show that VEGF does not represent a useful tool for early diagnosis of ovarian cancer. At 363.7 pg $ml^{-1}$, VEGF achieved a sensitivity of 54% and a specificity of 77% in our investigation (Figure 2A). With respect to differential diagnosis CA 125 revealed a sensitivity of 85% and a specificity of 93% at 74.9 U $ml^{-1}$ cut-off value. These excellent results might partly be influenced by the fact that our ovarian cancer population mainly consisted of advanced-stage patients. However, CA 125 levels have been repeatedly reported to be a useful diagnostic tool to differentiate benign from malignant ovarian masses (Markowska et al, 1994; Woolas et al, 1995; Gadducci et al, 1996).

While a strong correlation between CA 125 and tumour stage was found (data not shown), no correlation was detected between serum VEGF and the stage of disease in our study. This might indicate that CA 125 but not VEGF may represent a late symptom in tumour development and tumour growth. Among the 44 ovarian cancer patients, seven presented with stage I disease. Serum CA 125 levels were 22.0 U $ml^{-1}$ in two patients and higher than the usually used cut-off value of 35 U $ml^{-1}$ in the other five patients. In those two patients with low CA 125 levels, the corresponding VEGF values were 105.4 pg $ml^{-1}$ and 180.3 pg $ml^{-1}$, thus also remaining at a relatively low level. The present data therefore indicate that VEGF is only weakly excreted into the serum of ovarian cancer patients.

Although higher VEGF values were observed in ovarian cancer patients compared with patients with benign ovarian cysts, the serum VEGF level did not allow prediction of malignancy. The reason might be a large overlap of serum VEGF levels in patients with ovarian cancer and in those who had surgery for a benign cyst. This reasonable overlap can be explained by the fact that a significant proportion of patients with benign cysts also expressed VEGF at a high concentration. Another likely reason for the failure of VEGF to match usefulness of CA 125 in the diagnosis of ovarian cancer might be that CA 125 is largely a product of ovarian cancer cells, whereas VEGF is a protein made during a variety of physiological and non-physiological processes. VEGF is therefore likely to provide a high background.

Interpreting of serum VEGF levels also depends on the immunological methods used. The manufacturer of the kit used in this study claims that the assay was optimized to limit the amount of interference by non-specific factors in serum. No significant interference or cross-reactivity with various cytokines was found. However, so far alpha-2-macroglobulin and other serum proteins have not yet been tested, a possible interference resulting in epitope masking can not be completely excluded (Soker et al, 1993). The use of antibodies directed against N-terminus and C-terminus synthethic oligopeptides may result in false-negative results in the presence of proteolytical processes or degradation of VEGF (Yeo et al, 1992). As a result, N- or C-terminus-specific antibodies may fail to recognize VEGF. These potential drawbacks may be prevented by the use of polyclonal antibodies directed against human VEGF or bacterially expressed VEGF polypeptides. (Kondo et al, 1994). The immunoassay applied for the aim of this study uses antibodies raised against insect cell SF21-expressed recombinant human $VEGF_{165}$ reacting with naturally occurring human VEGF in a comparable matter. The kit manufacturer reports an average recovery rate of 102% from spiked serum. Considering all characteristics of this kit, the possible influence of VEGF degradation or complexing with serum proteins should be low.

Undoubtedly, VEGF plays an essential role in the growth and metastases of ovarian cancer. Boocock et al (1995) demonstrated that mRNA encoding VEGF, as well as VEGF receptors flt and KDR were detected in primary ascitic cells and in three out of four ovarian carcinoma cell lines by reverse transcriptase–polymerase chain reaction. By means of in situ hybridization, elevated expression of VEGF mRNA could be found in all primary tumours and their metastases. Recently, Mattern et al (1997) found a significant positive correlation of VEGF mRNA expression in ovarian carcinoma tissue and tumour cell proliferation rates as assessed by histone H3 mRNA expression. However, no association was found between the patients' clinical course and the extent of VEGF mRNA expression.

Although, there is abundant evidence to show that VEGF plays a central role in the development and the growth of malignant tumours, only scarce information is provided regarding serum VEGF levels in tumour patients. Recently, Takano et al (1996) reported about concentrations of VEGF in the serum and tumour tissue of brain tumour patients. No difference in serum VEGF concentration was found between nine patients with malignant brain tumours of different histological types with a median serum VEGF concentration of 40.4 pg $ml^{-1}$ (range 18.4–277.0 pg $ml^{-1}$) and healthy volunteers (range 28.4–184.4 pg $ml^{-1}$). Furthermore, no correlation was found between VEGF concentrations in serum and tumour extracts. In contrast, Yeo et al (1993) found elevated levels in malignant effusions, but failed to detect VEGF in the serum and urine obtained from patients with and without malignant ascites. They explained the failure to detect VEGF in the serum of malignant tumour patients by the clearance of VEGF from the circulation. In fact, the serum levels of a tumour marker are influenced by the balance of the tumour markers released into the effluent blood and its metabolism and excretion. As the half-life of VEGF is known to be only a few hours, one possible explanation for relatively low levels in patients with malignant tumours could be that VEGF is rapidly metabolized and excreted. To our knowledge, little is known about the pharmacokinetic or pharmacodynamic characteristics of VEGF.

Angiogenesis is normally seen in the female reproductive system as part of ovulation, endometrial proliferation, and the development and growth of the corpus luteum (Christenson and Stouffer, 1996). Kamat et al (1995) found VEGF mRNA and protein to be expressed by human ovarian granulosa and theca cells late in follicle development and subsequent to ovulation. By using the immunohistochemical approach, Gordon et al (1996) found intense VEGF immunostaining within the highly vascularized corpora lutea. They concluded that VEGF might contribute to fluid formation in ovarian cysts and that it plays an important role

© Cancer Research Campaign 1998

in the growth and maintenance of the ovarian follicle and corpus luteum by mediating angiogenesis. These data may indicate that VEGF is expressed during ovarian cyst formation as well as in neoplastic transformation.

In conclusion, our data demonstrate that VEGF may reflect tumour burden in patients with ovarian cancer. Because of the low sensitivity and specificity, VEGF does not seem to be a clinically useful discriminator between the absence or the presence of malignancy in patients who are intending to undergo surgery for suspected ovarian cancer.

### ACKNOWLEDGEMENTS

The authors would like to thank Ingrid Schiebel for performing the immunoassays. The present paper was supported by a grant from the 'Jubiläumsfonds der Österreichischen Nationalbank' (grant no. 5881) and by a grant from the 'Medizinisch-Wissenschaftlicher Fonds des Bürgermeisters der Bundeshauptstadt Wien'.

### REFERENCES

Bancher-Todesca D, Obermair A, Bilgi S, Kohlberger P, Kainz C, Breitenecker G, Leodolter S and Gitsch G (1997) Angiogenesis in vulvar intraepithelial neoplasia. *Gynecol Oncol* **64**: 496–500

Boocock CA, Charnock-Jones DS, Sharkey AM, McLaren J, Barker PJ, Wright KA, Twentyman PR and Smith SK (1995) Expression of vascular endothelial growth factor and its receptors flt and KDR in ovarian carcinoma. *J Natl Cancer Inst* **87**: 506–516

Campbell MJ and Machin D (1995) *Medical Statistics – a Commonsense Approach.* John Wiley & Sons: Chichester

Christenson LK and Stouffer RL (1996) Proliferation of microvascular endothelial cells in the primate corpus luteum during the menstrual cycle and simulated early pregnancy. *Endocrinology* **137**: 367–374

Ferrara N and Henzel WJ (1989) Pituitary follicular cells secrete a novel heparin-binding growth factor specific for vascular endothelial cells. *Biochem Biophys Res Commun* **161**: 851–859

Folkman J (1995) Angiogenesis in cancer, vascular, rheumatoid and other disease. *Nature Med* **1**: 27–31

Gadducci A, Baicchi U, Marrai R, Ferdeghini M, Bianchi R and Facchini V (1996) Preoperative evaluation of D-dimer and CA 125 levels in differentiating benign from malignant ovarian masses. *Gynecol Oncol* **60**: 197–202

Gordon JD, Mesiano S, Zaloudek CJ and Jaffe RB (1996) Vascular endothelial growth factor localization in human ovary and fallopian tubes: possible role in reproductive function and ovarian cyst formation. *J Clin Endocrinol Metab* **81**: 353–359

Guidi AJ, Abu-Jawdeh G, Berse B, Jackman RW, Tognazzi K, Dvorak HF and Brown LF (1995) Vascular permeability factor (vascular endothelial growth factor) expression and angiogenesis in cervical neoplasia. *J Natl Cancer Inst* **87**: 1237–1245

Hosmer DJ and Lemeshow S (1989) *Applied Logistic Regression.* Wiley: New York

International Federation of Gynecologists and Obstetricians (FIGO) (1987) Changes in definitions of clinical staging for carcinoma of the cervix and ovary. *Am J Obstet Gynecol* **156**: 263–264

Jacobs I and Bast RC (1989) The CA 125 tumor-associated antigen: a review of the literature. *Hum Reprod* **4**: 1–12

Jacobs IJ, Skates S, Davies AP, Woolas AP, Jeyerajah A, Weidemann P, Sibley K and Oram DH (1996) Risk of diagnosis of ovarian cancer after raised serum CA 125 concentration: a prospective cohort study. *Br Med J* **313**: 1355–1358

Kamat BR, Brown LF, Manseau EJ, Senger DR and Dvorak HF (1995) Expression of vascular permeability factor/vascular endothelial growth factor by human granulosa and theca lutein cells. *Am J Pathol* **146**: 157–165

Ke-Lin, Qu-Hong, Nagy JA, Eckelhoefer IA, Masse EM, Dvorak AM and Dvorak HF (1996) Vascular targeting of solid and ascites tumours with antibodies to vascular endothelial growth factor. *Eur J Cancer* **32A**: 2467–2473

Klauber N, Rohan RM, Flynn E and D'Amato RJ (1997) Critical components of the female reproductive pathway are suppressed by the angiogenesis inhibitor AGM-1470. *Nature Med* **3**: 443–446

Kondo S, Asano M, Matsuo K, Ohmori I and Suzuki H (1994) Vascular endothelial growth factor/vascular permeability factor is detectable in the sera of tumor-bearing mice and cancer patients. *Biochim Biophys Acta* **1221**: 211–214

Markowska J, Kopczynski Z, Szewierski Z, Markowski M and Niecewicz R (1994) The value of estimating CA 125 in fluids from benign or malignant cysts, in the exudate and blood serum in women with ovarian cancer. *Eur J Gynaecol Oncol* **15**: 29–32

Mattern J, Stammler G, Koomagi R, Wallwiener D, Kaufmann M and Volm M (1997) Association of vascular endothelial growth factor expression with tumor cell proliferation in ovarian carcinoma. *Anticancer Res* **17**: 621–624

Melnyk O, Shuman MA and Kim KJ (1996) Vascular endothelial growth factor promotes tumor dissemination by a mechanism distinct from its effect on primary tumor growth. *Cancer Res* **56**: 921–924

Neufeld G, Cohen T, Gitay-Goren H, Poltorak Z, Tessler S, Sharon R, Gengrinovitch S and Levi BZ (1996) Similarities and differences between the vascular endothelial growth factor (VEGF) splice variants. *Cancer Metas Rev* **15**: 153–158

Obermair A, Bancher-Todesca D, Bilgi S, Kaider A, Kohlberger P, Müllauer-Ertl S, Leodolter S and Gitsch G (1997) Correlation of vascular endothelial growth factor expression and microvessel density in cervical intraepithelial neoplasia. *J Natl Cancer Inst* **89**: 1212–1217

Oram DH and Jeyarajah AR (1994) The role of ultrasound and tumor markers in the early detection of ovarian cancer. *Br J Obstet Gynaecol* **101**: 939–945

Senger DR, Galli SJ, Dvorak AM, Peruzzi CA, Harvey VS and Dvorak HF (1983) Tumor cells secrete a vascular permeability factor that promotes accumulation of ascites fluid. *Science* **219**: 983–985

Serov SF, Scully RE and Sarbin LH (1973) *International Histological Classification of Tumors.* WHO: Geneva

Soker S, Svahn S and Neufeld G (1993) Vascular endothelial growth factor is inactivated by binding to alpha 2-macroglobulin and the binding is inhibited by heparin. *J Biol Chem* **268**: 7685–7691

Takano S, Yoshii Y, Kondo S, Suzuki H, Maruno T, Shirai S and Nose T (1996) Concentration of vascular endothelial growth factor in the serum and tumor tissue of brain tumor patients. *Cancer Res* **56**: 2185–2190

Terman BI and Dougher-Vermazen M (1996) Biological properties of VEGF/VPF receptors. *Cancer Metast Rev* **15**: 159–163

Woolas RP, Conaway MR, Xu F, Jacobs IJ, Yu Y, Daly L, Davies AP, O'Briant K, Berchuck A, Soper JT, Clarke-Pearson DL, Rodriguez G, Oram DH and Bast RC Jr (1995) Combinations of multiple serum markers are superior to individual assays for discriminating malignant from benign pelvic masses. *Gynecol Oncol* **59**: 111–116

Yeo KT, Sioussat TM, Faix JD, Senger DR and Yeo TK (1992) Development of time-resolved immunofluorometric assay of vascular permeability factor. *Clin Chem* **38**: 71–75

Yeo KT, Wang HH, Nagy JA, Sioussat TM, Ledbetter SR, Hoogewerf AJ, Zhou Y, Masse EM, Senger DR, Dvorak HF and Yeo TK (1993) Vascular permeability factor (vascular endothelial growth factor) in guinea pig and human tumor and inflammatory effusions. *Cancer Res* **53**: 2912–2918

*© Cancer Research Campaign 1998*